# *Rsk2* Knockdown in PC12 Cells Results in Sp1 Dependent Increased Expression of the *Gria2* Gene, Encoding the AMPA Receptor Subunit GluR2

**DOI:** 10.3390/ijms14023358

**Published:** 2013-02-06

**Authors:** Tahir Mehmood, Anne Schneider, Solange Pannetier, André Hanauer

**Affiliations:** 1 Institut de Génétique et de Biologie Moléculaire et Cellulaire (IGBMC), Department of Translational Medicine and Neurogenetics, INSERM U 964, CNRS UMR 1704, Université de Strasbourg, 67404 Illkirch, France; E-Mails: schneider.anne@hotmail.fr (A.S.); solange@igbmc.fr (S.P.); 2 Department of Chemistry, University of Sargodha, 40100 Sargodha, Pakistan

**Keywords:** Coffin-Lowry syndrome, ERK pathway, RSK2, GluR2, Sp1

## Abstract

The RSK2 protein is a member of the RSK serine-threonine protein kinase family and is encoded by the X-linked *rps6ka3* gene in human. Highly heterogeneous loss-of-function mutations affecting this gene are responsible for a severe syndromic form of cognitive impairment, Coffin-Lowry syndrome. RSK2, which is highly conserved in mammals, acts at the distal end of the Ras-ERK signaling pathway and is activated in response to growth factors and neurotransmitters. RSK2 is highly expressed in the hippocampus, and *Rsk2*-KO mice display spatial learning and memory impairment. We recently showed that ERK1/2 activity is abnormally increased in the hippocampus of *Rsk2*-KO mice as well as the expression of the AMPA receptor subunit GluR2. The mechanism via which RSK2 deficiency affects the expression of GluR2 in neural cells was unknown. To address this issue we constitutively suppressed the expression of RSK2 in PC12 cells via vector-based shRNA in the present study. We show that *Rs*k2 silencing leads also to an elevation of ERK1/2 phosphorylation as well as of GluR2 expression and that the increased level of GluR2 expression results from the increased ERK1/2 activity on the transcription factor Sp1. Our results provide evidence that RSK2 modulates ERK1/2 activity on Sp1, which regulates GluR2 expression through transcriptional activation.

## 1. Introduction

The 90 kDa ribosomal S6 kinases (RSKs) constitute a family of four homologous Ser/Thr kinases (RSK1-4) that are activated by Mitogen activated protein kinase/extracellular-regulated kinases 1 and 2 (MAPK/ERK1/2) in response to growth factors, hormones, chemokines and neurotransmitters. When activated by Extracellular signal-regulated kinase (ERK), a fraction of RSK2 in turn phosphorylates a number of cytoplasmic substrates such as BCL2-associated agonist of cell death (Bad), p53, L1 cell adhesion molecule (L1CAM) and Glycogen synthase kinase 3 (GSK3) [1]. Another fraction of RSK2 translocates into the nucleus where it is thought to regulate gene expression through phosphorylation of transcription factors such as cAMP-response element binding protein (CREB), the cellular FBJ murine osteosarcoma viral oncogene homolog (c-fos), the oestrogen nuclear receptor, the nuclear receptor subfamily 77 (Nurr 77) and the Activating transcription factor 4 (ATF4) [1,2]. The roles of RSKs in cellular signaling, cell survival, growth and differentiation has been well established; however, their implication in biological processes *in vivo* is less well known. An important physiological role of one RSK member, RSK2, was uncovered by the discovery of *Rsk2* gene defects in the Coffin-Lowry Syndrome (CLS) [3]. CLS is an X-linked disorder with progressive skeletal abnormalities, facial dimorphism, and strong psychomotor impairment [4]. The vast majority of male CLS patients show severe cognitive deficiency with intelligence quotient (IQ) ranging from 15 to 60. Delay in speech acquisition is particularly common with most affected males having a very limited vocabulary. The mouse knockout (KO) model (*Rsk2*-KO) for CLS exhibits a severe impairment in spatial learning and a deficit in long-term spatial memory [5]. Moreover, in human and mouse brain, RSK2 is highly expressed in the hippocampus, an essential brain structure in cognitive function and learning [6,7]. Together the data suggest that RSK2 plays an important role in cognitive function in human and in mice.

To gain greater insight into the molecular mechanisms leading to learning and memory impairments in the *Rsk2*-KO mice and to intellectual disability in CLS, we examined recently global gene expression profiles in hippocampus from KO mice, It revealed altered expression of 100 genes encoding proteins acting in various biological pathways [8]. We investigated in detail the consequences of the two-fold up-regulation of one of these genes, Glutamate receptor, ionotropic *2* (*Gria2*), which encodes the subunit GluR2 of the alpha-amino-3-hydroxy-5-methyl-4-isoxazolepropionic acid (AMPA) receptor. Abnormally increased expression of GluR2 at the protein level was confirmed in the hippocampus of *Rsk2*-KO mice and at synapses from *Rsk2*-KO hippocampus primary neuron cultures. Basal AMPA receptor-mediated activity was found to be significantly decreased [8]. RSK2 deficient mice have also an increased baseline level of phospho-ERK1/2 as well as enhanced activation of ERK1/2 after glutamate stimulation of hippocampal neurons [9]. These latter findings definitively demonstrated that RSK2 exerts a feedback inhibitory effect on the ERK1/2 pathway in physiological conditions.

To define the molecular events leading to GluR2 over expression in RSK2 deficient neurons we used in the present study the RNA interference technology to knockdown the *Rsk2* gene in PC12 cells. We show that *Rs*k2 silencing in PC12 cells leads also to an increase of ERK1/2 activity and of GluR2 expression at the mRNA and protein levels. Our data reveal that the increased GluR2 expression results from the increased ERK1/2 activity on the transcription factor Specificity Protein 1 (Sp1).

## 2. Results and Discussion

### 2.1. *Rsk2* Knockdown of PC12 Cells

**Figure 1:**
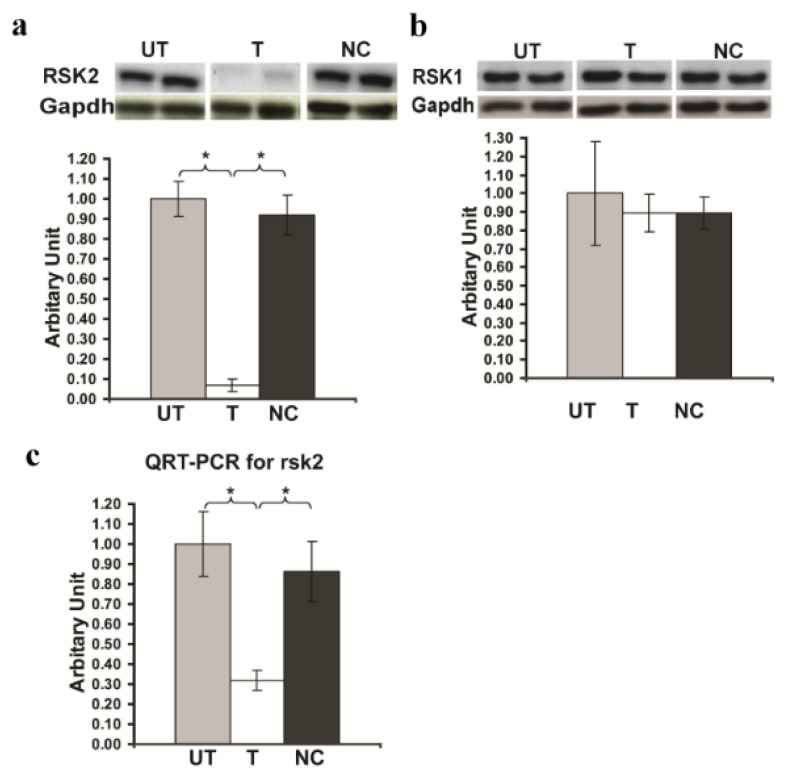
Silencing of the *Rsk2* gene in PC12 cells. (**A**) Detection of the RSK2 protein level in Untransfected (UT), transfected with the *Rsk2*-shRNA (T) or the scrambled-shRNA (negative control, NC) PC12 cells. Data normalized to glyceraldehyde-3-phosphate dehydrogenase (GAPDH) are represented as the mean values ± SEM (UT: *n* > 8, T: *n* > 8, NC: *n* > 8). RSK2 expression is drastically decreased in *Rsk2*-shRNA transfected cells (*p < 0.05 by Student’s *t*-test). (**B**) RSK1 protein expression in PC12 cells. Western blots results showed no significant difference between UT, T and NC cells (UT: *n* > 3, T: *n* > 3, NC: *n* > 3). (**C**) Quantification of the expression level of the *Rsk2* mRNA by real time QRT-PCR (T: *n* > 3, UT: *n* > 3, NC: *n* > 3). Data are normalized to the *Gapdh* gene expression. Bar represents the mean ± SEM. *p < 0.05 by Student’s *t*-test.

For this study we used the PC12 cell line, an immortalized cell line derived from a pheochromocytoma tumor of the rat adrenal gland. NGF-treated PC12 cells cease proliferation, grow long neurites, and show changes in cellular composition associated with neuronal differentiation [10], and can therefore serve as a model system for primary neuronal cells. PC12 cells express of RSK2 as shown in (Figure 1). We tested the efficacy to block RSK2 expression in PC12 cells of three plasmids producing different RSK2-specific small hairpin RNAs (*Rsk2*-shRNA) targeted to different coding regions of the mRNA of RSK2. The recombinant plasmids were transfected into the PC12 cells by lipofectamine 2000, recombinant cells selected 72 h on G418, and mRNA and protein expression levels of the *Rsk2* gene were assayed by QRT-PCR and Western blot. One shRNAs (shRNA clone ID7) decreased *Rsk2* gene expression dramatically (Figure 1a). Quantification of the immunoblots revealed a 90% reduction in the expression of RSK2 protein, compared to untransfected PC12 cells. No significant change was found in the level of RSK2 mRNA and protein expression in cells transfected with a shRNA containing a scrambled sequence (negative control) (Figure 1a). (Expression of RSK2 protein in *Rsk2*-shRNA transfected cells: 0.07 ± 0.03; untransfected cells: 1.0 ± 0.09; scrambled-shRNA transfected cells: 0.917 ± 0.1; *p < 0.05 by Student’s *t*-test) (Figure 1a). QRT-PCR revealed also a dramatic decrease of RSK2 mRNA expression (Expression of RSK2 mRNA in *Rsk2*-shRNA transfected cells: 0.316 ± 0.05; untransfected cells: 1.0 ± 0.16; scrambled-shRNA transfected cells; 0.865 ± 0.15; *p < 0.05 by Student’s *t*-test) (Figure 1c). Thus, the shRNA clone ID7 was used in all subsequent studies. We then asked whether the severe down regulation of RSK2 would also affect the expression of other RSK proteins in PC12 cells. The result for RSK1 is shown in Figure 1b. (Expression of RSK1 protein in *Rsk2*-shRNA transfected cells: 0.895 ± 0.1; untransfected cells: 1.0 ± 0.28; scrambled-shRNA transfected cells: 0.894 ± 0.09; *p < 0.05 by Student’s *t*-test). RSK3 was very weakly expressed in untransfected PC12 cells and was unchanged in transfected PC12 cells (result not shown). Thus, none of these two latter RSK family members apparently exhibited any significant change in its expression levels, documenting that the *Rsk2*-shRNA approach was highly specific for RSK2 and reducing the possibility of compensation for the RSK2 loss via over expression of other RSK family members.

### 2.2. Immunohistochemical Analysis of RSK2

**Figure 2:**
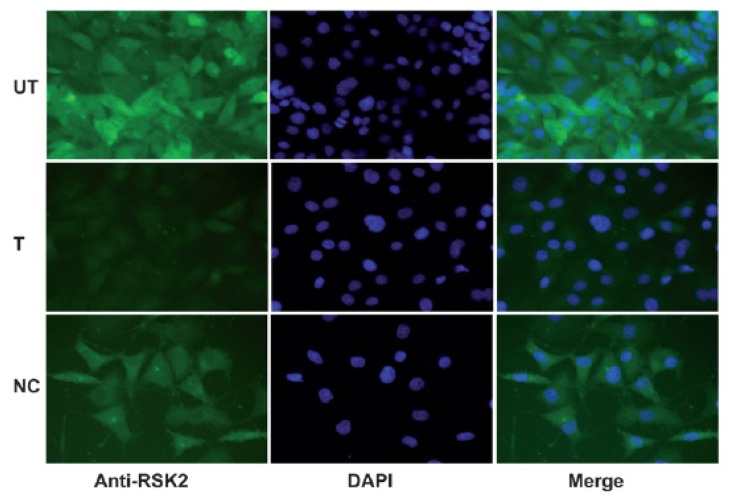
Immunocytofluorescence studies. RSK2 expression in untransfected (UT), transfected with the *Rsk2*-shRNA (T) or the scrambled-shRNA (negative control, NC) PC12 cells. The anti-RSK2 antibody reveals a strong RSK2 staining in UT and NC cells, whereas the signal is very faint in *Rsk2*-shRNA transfected (T) cells, confirming the dramatically decreased expression of RSK2 in *Rsk2*-shRNA transfected cells (UT: *n* > 3, T: *n* > 3, NC: *n* > 3). DAPI: 4′,6′-diamidino-2-phénylindole.

We also examined the expression of RSK2 in PC12 cells by immunohistochemical staining. Most of untransfected, or scrambled-shRNA transfected PC12 cells showed strong RSK2 staining, whereas *Rsk2*-shRNA transfected cells expressed RSK2 very weakly (Figure 2). It was concluded that the *Rsk2*-shRNA recombinant plasmid can effectively suppress the expression of the *Rsk2* gene in PC12 cells.

### 2.3. Rsk2 Silencing Increased ERK1/2 MAPK Phosphorylation

**Figure 3:**
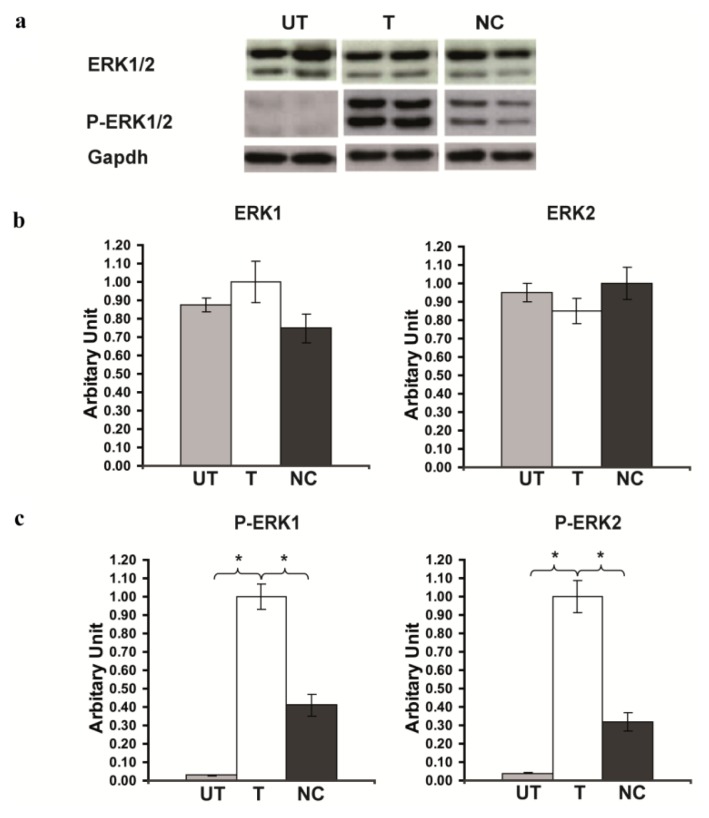
Strong increase of Phospho-ERK1/2 (P-ERK1/2) activity in RSK2 depleted PC12 cells. (**A**) Western blot analysis of the ERK1/2 and P-ERK1/2 protein levels in untransfected (UT), transfected with the *Rsk2*-shRNA (T) and scrambled-shRNA (negative control, NC) PC12 cells. The results revealed a much higher level of P-ERK1/2 in RSK2 depleted PC12 cell than in the other conditions. (**B**) Quantification of ERK1 and 2 normalized to GAPDH revealed no significant difference between the various conditions. (**C**) Quantification of P-ERK1 and 2 expressions, normalized to ERK1 or 2, respectively. The levels were significantly higher in *Rsk2*-shRNA transfected (T) cells than in the other conditions. Data are represented as the mean ± SEM (UT: *n* > 6, T: *n* > 6, NC: *n* > 6, *p <0.05 by Student’s *t*-test).

We then asked whether *Rsk2* knockdown affects the level of ERK1/2 phosphorylation in PC12 cells. Detection of total ERK levels revealed no significant difference of ERK protein expression between untransfected PC12 cells and cells transfected with the *Rsk2*- or scrambled-shRNA (Expression of ERK1 protein in *Rsk2*-shRNA transfected cells: 1.0 ± 0.11; untransfected cells: 0.875 ± 0.04; scrambled-shRNA transfected cells: 0.748 ± 0.08, Expression of ERK2 protein in *Rsk2*-shRNA transfected cells: 0.830 ± 0.07; untransfected cells: 0.950 ± 0.05; scrambled-shRNA transfected cells: 1.0 ± 0.09; *p < 0.05 by Student’s *t*-test) (Figure 3a,b). In contrast, the analysis of phosphorylated ERK1 and ERK2 showed that the active forms of both ERK1 (P-ERK1) and ERK2 (P-ERK2) were significantly higher in *Rsk2*-shRNA transfected PC12 cells than in scrambled-shRNA transfected or untransfected cells (Expression of P-ERK1 in *Rsk2*-shRNA transfected cells: 1.0 ± 0.07; untransfected cells: 0.03 ± 0.003; scrambled-shRNA transfected cells: 0.42 ± 0.06, Expression of P-ERK2 in *Rsk2*-shRNA transfected cells: 1.0 ± 0.09; untransfected cells: 0.04 ± 0.003; scrambled-shRNA transfected cells: 0.32 ± 0.05; *p <0.05 by Student’s *t*-test) (Figure 3 a,c). These results were consistent with our previous data in the hippocampus of *Rsk2*-KO mice, which also showed increased levels of ERK1/2 phosphorylation [9]. A much smaller increase of P-ERK1/2 levels was also observed in scrambled-shRNA transfected PC12 cells. The cause is not yet known but it might be due to the G418 selection.

### 2.4. *Rsk2* Knockdown Results in up Regulation of *Gria2* Gene (Encoding GluR2) Expression

**Figure 4:**
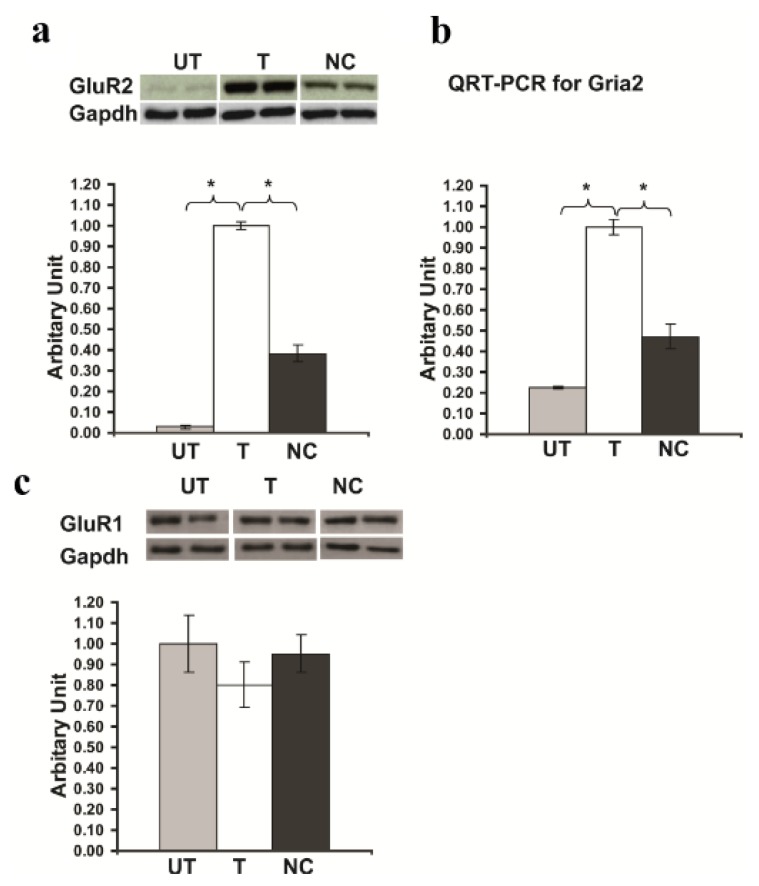
Quantitative analysis of GluR2 protein and *Gria2* gene expression. Untransfected (UT), transfected with the *Rsk2*-shRNA (T) or the scrambled-shRNA (negative control, NC) PC12 cells. (**A**) The GluR2 protein expression level is much higher in *Rsk2*-shRNA transfected cells as compared with (UT) and (NC). Data, normalized to GAPDH expression, are represented as the mean ± SEM (UT: *n* > 6, T: *n* > 6, NC: *n* > 6, *p < 0.05 by Student’s *t*-test. (**B**) Quantification of the expression level of the *Gria2* mRNA by real time QRT-PCR and normalized to the *Gapdh* gene expression. A significantly higher expression of the *Gria2* mRNA was observed in *Rsk2*-shRNA (T) transfected cells, than in UT or NC PC12 cells (UT: *n* > 3, T: *n* > 3, NC: *n* > 3). Bar represents the mean ± SEM *p < 0.05 by Student’s *t*-test. (**C**) Quantitative analysis of GluR1 protein expression. After normalization with GAPDH, no significant difference was observed between the three conditions (UT: *n* > 3, T: *n* > 3, NC: *n* > 3).

We next examined the expression of GluR2 in *Rsk2* knockdown PC12 cells by Western blot analysis and QRT-PCR. Detection of GluR2 protein levels, revealed a very strong increase of expression in *Rsk2* knockdown PC12 cells (Expression of GluR2 protein in *Rsk2*-shRNA transfected cells: 1.0 ± 0.02; untransfected cells: 0.03 ± 0.008; scrambled-shRNA transfected cells: 0.382 ± 0.04; *p < 0.05 by Student’s *t*-test) (Figure 4a). QRT-PCR revealed also an approximately four-fold higher *Gria2* mRNA expression in *Rsk2*-silenced PC12 cells compared to untransfected cells (Expression of *Gria2* mRNA in *Rsk2*-shRNA transfected cells: 1.0 ± 0.04; untransfected cells: 0.225 ± 0.007; scrambled-shRNA transfected cells: 0.476 ± 0.06; *p < 0.05 by Student’s *t*-test) (Figure 4b). Cells transfected with the scrambled-shRNA showed a small increase of both GluR2 protein and mRNA but much lower than *Rsk2*-shRNA transfected cells, and which may be related to the small increase of ERK1/2 phosphorylation observed in these cells (Figure 4 a,b). We assayed also GluR1 and GluR3 levels of expression. Detection of GluR1 protein levels revealed a small decrease of expression in *Rsk2*-shRNA transfected PC12 cells compared to untransfected PC12 cells or cells transfected with the scrambled-shRNA (Expression of GluR1 protein in *Rsk2*-shRNA transfected cells: 0.803 ± 0.11; untransfected cells: 1.0 ± 0.14; scrambled-shRNA transfected cells: 0.951 ± 0.09; *p < 0.05 by Student’s *t*-test) (Figure 4c). GluR3 was hardly detectable in untransfected PC12 cells as well as in *Rsk2*- and scrambled-shRNA transfected cells (not shown), suggesting that expression of GluR3 was not altered in RSK2 deficient cells. These results showed that only the expression of GluR2 is up regulated in response to RSK2 deficiency and suggested also that the regulatory mechanisms of expression are different for each GluR gene.

### 2.5. Inhibition of GluR2 over Expression in *Rsk2* Knockdown PC12 Cells by U0126

**Figure 5:**
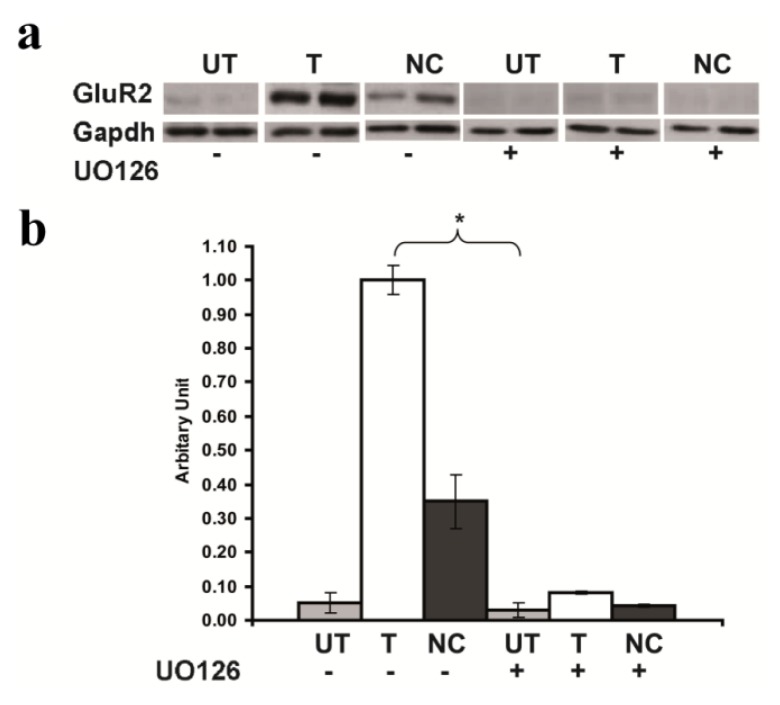
Application of the MEK inhibitor U0126 to untransfected (UT), transfected with *Rsk2*-shRNA (T) or with scrambled-shRNA (negative control, NC) PC12 cells. The level of expression of the GluR2 protein was dramatically decreased in the *Rsk2*-shRNA transfected PC12 cell line and treated with U0126 when compared to the same *Rsk2*-shRNA transfected cells but without U0126 treatment. Data are normalized to GAPDH and are represented as the mean ± SEM (UT: *n* > 4, T: *n* > 4, NC: *n* > 4, *p <0.05 by Student’s *t*-test).

We then evaluated if the increased level of active ERK may be the cause of the strong increase of GluR2 expression in *Rsk2* knockdown PC12 cells. To this end, *Rsk2*- and scrambled-shRNA transfected PC12 cells as well as untransfected cells were treated for 24 h, before being harvested, with U0126 (20 μM), a MEK inhibitor (MEK being the ERK1/2 upstream kinase in the ERK/MAPK pathway). U0126 dramatically inhibited the GluR2 up-regulation of expression induced by *Rsk2* silencing, as detected by Western blot analysis (Expression of GluR2, protein without U0126 treatment, in *Rsk2*-shRNA transfected cells: 1.0 ± 0.043; untransfected cells: 0.05 ± 0.03; scrambled-shRNA transfected cells: 0.35 ± 0.08; Expression of GluR2 protein after U0126 treatment in *Rsk2*-shRNA untransfected cells: 0.03 ± 0.02; transfected cells: 0.08 ± 0.004 and scrambled-shRNA transfected cells: 0.043 ± 0.005; *p < 0.05 by Student’s *t*-test) (Figure 5). These results suggested that ERK1/2 activity plays a crucial role in the up regulation of GluR2 expression induced by RSK2 depletion.

### 2.6. Involvement of Sp1 in the up Regulation of GluR2 Expression in *Rsk2* Knockdown PC12 Cells

**Figure 6:**
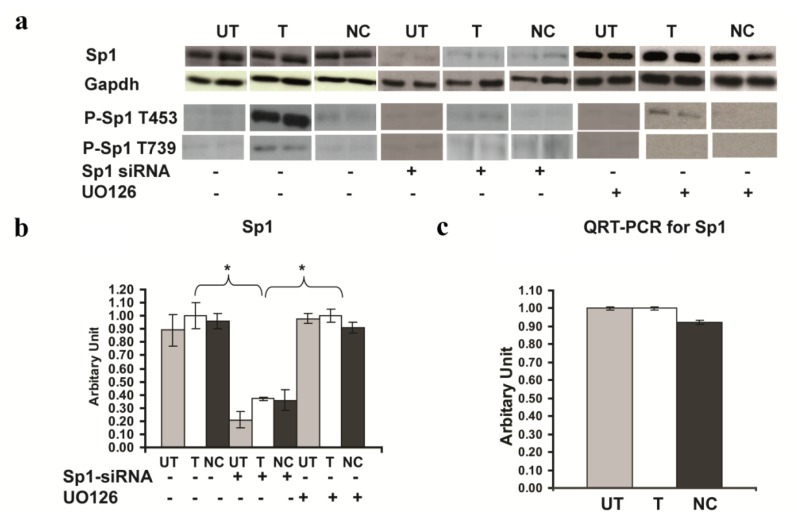

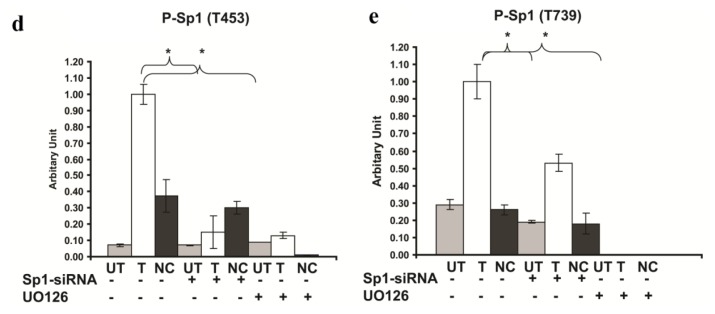
Expression levels of Sp1 and phospho-Sp1 (P-Sp1) in untransfected (UT), transfected with the *Rsk2*-shRNA (T) or the scrambled-shRNA (negative control, NC) PC12 cells. (**A**) Western blot results. (**B**) Quantification of Sp1 protein expression levels. Data, normalized with GAPDH, are represented as the mean ± SEM (UT: *n* > 3, T: *n* > 3, NC: *n* > 3). Sp1 protein expression is strongly decreased in Sp1-siRNA transfected cells, whereas in all other conditions it is very similar. (**C**) Quantification of Sp1 mRNA expression by QRT-PCR and normalized to the *Gapdh* gene expression (UT: *n* > 3, T: *n* > 3, NC: *n* > 3). Bar represents the mean ± SEM. *p < 0.05 by Student’s *t*-test. (**D**,**E**) Levels of P-Sp1 (T453 and T739) are much higher in *Rsk2*-shRNA transfected (T) cells as in all other conditions. Differences with *p < 0.05 between UT and T and NC groups were considered significant by Student’s *t*-test.

It was previously shown that GluR2 expression is strongly influenced at the transcriptional level by a positive Sp1 regulatory element in the 5′ proximal region of the *Gria2* gene promoter [11]. The transcription factor Sp1 recruits basal transcription factor TFIID to DNA and induces transcription. It was also reported that Sp1 activity increases when phosphorylated by ERK at two specific threonine residues (Thr453 and Thr739) [12]. Thus, we wondered whether the expression and/or the phosphorylation of Sp1 are also up regulated in *Rsk2* knockdown PC12 cells. Total protein was extracted for analysis of Sp1 and Phospho-Sp1 (P-Sp1) expression by Western blotting. Total Sp1 levels, revealed no difference of Sp1 mRNA and protein expression between untransfected and *Rsk2*-shRNA transfected PC12 cells (mRNA expression of Sp1 in *Rsk2*-shRNA transfected cells: 1.0 ± 0.009; untransfected cells: 1.0 ± 0.006; scrambled-shRNA transfected cells: 0.921 ± 0.01; expression of Sp1 protein in *Rsk2*-shRNA transfected cells: 1.0 ± 0.12; untransfected cells: 0.89 ± 0.1; scrambled-shRNA transfected cells: 0.961 ± 0.06 *p < 0.05 by Student’s *t*-test) (Figure 6a–c). However, immunoblot analysis of phosphorylated forms of Sp1 showed that the levels of phosphorylation at both phosphorylation sites, T453 and T739, were significantly higher in *Rsk2-shRNA* transfected cells than in untransfected cells (Expression of P-Sp1 (T453) in *Rsk2*-shRNA transfected cells: 1.0 ± 0.06; untransfected cells: 0.07 ± 0.01; scrambled-shRNA transfected cells: 0.376 ± 0.1; Expression of P-Sp1 (T739) in *Rsk2*-shRNA transfected cells: 1.0 ± 0.1; untransfected cells: 0.29 ± 0.03; scrambled-shRNA transfected cells: 0.26 ± 0.03; *p < 0.05 by Student’s *t*-test (Figure 6 d,e).

To further determine whether treatment with the MEK inhibitor U0126 impacts Sp1 phosphorylation, *Rsk2*-shRNA transfected and untransfected PC12 cells were treated with this inhibitor (20 μM) for 24 h before being harvested. Total proteins were extracted for Western blot analysis and Sp1 protein levels, revealed no difference between *Rsk2*-transfected, untransfected and scrambled cells (expression of Sp1 in *Rsk2*-shRNA untransfected: 0.91 ± 0.04 transfected cells: 1.0 ± 0.05; scrambled shRNA transfected cells: 0.98 ± 0.03). (Figure 6 a,b) Inhibition of ERK1/2 activity resulted in a dramatic decrease of Sp1 phosphorylation at both T739 and T453 sites (Expression of P-Sp1 (T453) after U0126 treatment in *Rsk2*-shRNA untransfected cells: 0.09 ± 0.002; transfected cells 0.13 ± 0.02; scrambled-shRNA transfected cells: 0.001 ± 0.005). Expression of P-Sp1 (T739) after U0126 treatment was undetectable (Figure 6 a,e). These results demonstrated that ERK is the major kinase that phosphorylates Sp1 at both of these sites.

**Figure 7:**
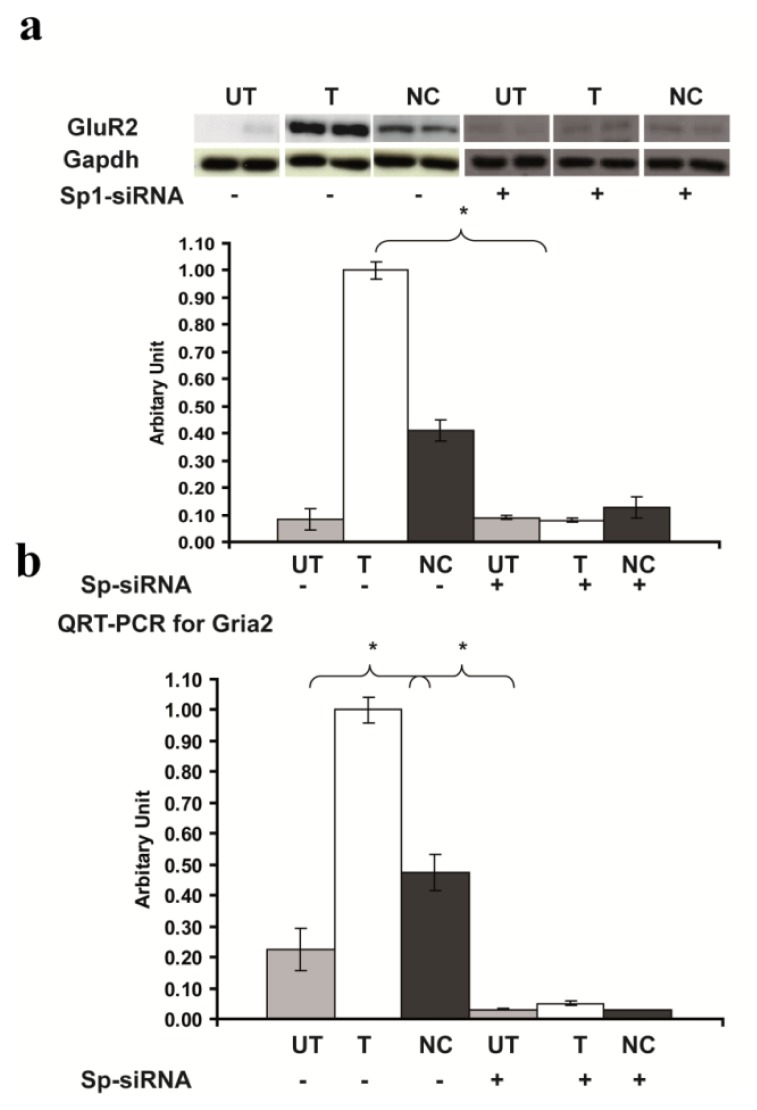
GluR2 expression is dramatically decreased in *Rsk2* knockdown cells treated with the Sp1-siRNA. (**A**) Western blot results revealed a strong decrease of GluR2 expression in Sp1 silenced *Rsk2* knockdown PC12 cell, as compared to *Rsk2* knockdown cells without Sp1 silencing. (**B**) Quantification of *Gria2* mRNA expression by QRT-PCR. Data are normalized to *Gapdh* mRNA expression and are represented as the mean ± SEM (UT: *n* > 3, T: *n* > 3, NC: *n* > 3) (*p < 0.05 by Student’s *t*-test).

To further confirm the crucial role of Sp1 in the up-regulation of *Gria2* expression, we down-regulated partially the expression of Sp1 by RNAi. Forty-eight hours after transfection, cells were harvested, total protein extracted and Western blotting performed. Sp1-siRNA reduced by about two-thirds the levels of Sp1 protein expression in *Rsk2*-shRNA transfected PC12 cells (Expression of Sp1 protein after Sp1-siRNA treatment in untrasfected 0.20 ± 0.06, *Rsk2*-shRNA transfected cells: 0.37 ± 0.01; scrambled-shRNA transfected cells: 0.36 ± 0.08) (Figure 6 a,b). The level of GluR2 expression was also much lower in Sp1-siRNA transfected *Rsk2* knockdown PC12 cells as well as in *Rsk2* knock down PC12 cells that are not transfected with Sp1-siRNA, and similar to untransfected PC12 cells (Expression of GluR2 protein before Sp1-siRNA transfection in *Rsk2*-shRNA transfected: 1.0 ± 0.03; untransfected cells: 0.084 ± 0.04; scrambled-shRNA transfected cells: 0.341 ± 0.04; and after Sp1-siRNA transfection in *Rsk2*-shRNA transfected cells: 0.08 ± 0.008; untransfected: 0.09 ± 0.009 scrambled-shRNA transfected cells: 0.126 ± 0.04; *p < 0.05 by Student’s *t*-test). (Figure 7a). QRT-PCR revealed also a similar decrease of *Gria2* mRNA expression following Sp1-siRNA application (Expression of *Gria2* mRNA before Sp1-siRNA transfection in *Rsk2*-shRNA transfected cells: 1.0 ± 0.04; untransfected cells: 0.225 ± 0.07; scrambled-shRNA transfected cells: 0.475 ± 0.06; and after Sp1-siRNA transfection, *Rsk2*-shRNA transfected cells: 0.05 ± 0.008; untrnasfected: 0.03 ± 0.008 and scrambled-shRNA transfected cells: 0.03 ± 0.001; *p < 0.05 by Student’s *t*-test) (Figure 7b). These results further demonstrate that Sp1 is involved in the increased GluR2 expression in RSK2 deficient PC12 cells.

Together with previously reported data on regulation of the *Gria2* gene and Sp1 activity, our results show that an abnormally increased level of Sp1 phosphorylation in response to ERK1/2 over activation is responsible for up regulated transcription of the *Gria2* gene in RSK2 deficient PC12 cells.

### 2.7. Confirmation of Significantly Increased Sp1 Phosphorylation in the Hippocampus of *Rsk2*-KO Mice

**Figure 8:**
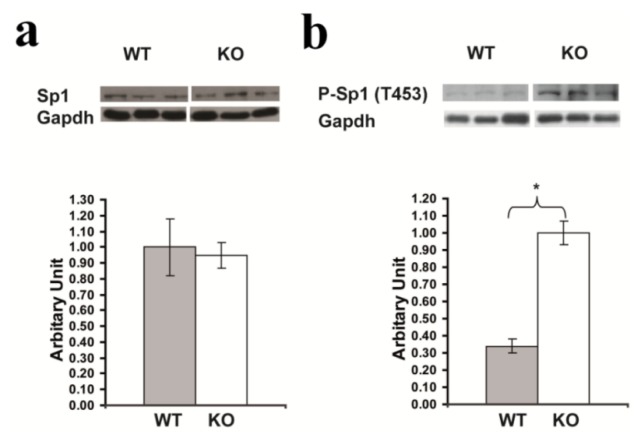
Sp1 and Phospho-Sp1 (P-Sp1) levels determined from hippocampus protein extracts of *Rsk2*-KO and WT littermate male mice. (**A**) Western Blot data and quantification of Sp1 expression levels normalized to GAPDH expression and represented as the mean values ± SEM (WT: *n* > 5; KO: *n* > 5). (**B**) Western blots results and quantification of P-Sp1 (T453) expression levels. Data are normalized to Sp1 protein expression and are represented as the mean values ± SEM (WT: *n* > 5; KO: *n* > 5). The level of phospho-Sp1 (T453) expression is significantly higher in *rsk2*-KO mice as in WT littermate. *p < 0.05 by Student’s *t*-test.

Western blot analysis of total protein extracts from hippocampi of *Rsk2-*KO adult mice also revealed significantly increased levels of P-Sp1 (T453) as compared to WT littermate. No significant difference was observed for Sp1 protein levels (Sp1: WT: 1.0 ± 0.18; KO: 0.95 ± 0.08; and P-Sp1 (T453): WT: 0.34 ± 0.04; KO: 1.0 ± 0.07) (Figure 8).

## 3. Discussion

The (AMPA)-type glutamate receptor channels are expressed ubiquitously in brain neurons and mediate fast excitatory neurotransmission in most excitatory synapses. AMPA receptors are assembled from combinations of four subunits, GluR1, GluR2, GluR3 and/or GluR4. AMPA receptors play also a crucial role in synaptogenesis and formation of neuronal circuitry, as well as in synaptic plasticity [13]. There is a huge amount of evidence indicating that the GluR2 subunit plays a pivotal role in AMPA receptors function. The Ca^++^ permeability, rectification, and single-channel conductance of AMPA receptors are all dominantly influenced by inclusion of an edited GluR2 subunit in the receptor complex [14–20]. Receptors that contain a single edited GluR2 subunit have maximally reduced Ca^++^ permeability, whereas inward rectification is reduced in a graded manner as the number of GluR2 subunits in a receptor increase [20]. Thus, moderate changes in GluR2 expression may have functional consequences on receptor subunit rearrangement and lead to functional differences in the functioning of synaptic circuits. GluR2 expression is governed by neuronal activity [21], and its synaptic distribution and levels are modulated as part of the mechanism of synaptic plasticity [22,23]. GluR2 interacts also with various molecules implicated in receptor trafficking [24–26]. The crucial role played by GluR2 in neuronal function may therefore explain why it is the most tightly regulated of the AMPA receptor subunits [26].

The two-fold increased level of GluR2 expression in *Rsk2*-KO mice prompted us to elucidate the molecular mechanism leading to this alteration of expression by silencing the *Rsk2* gene in PC12 cells. The PC12 cell line, cloned from rat pheochromocytoma cells, can be induced to differentiate and to acquire a neuronal-like phenotype [10]. PC12 cells express endogenous RSK2 at a relatively high level, whereas the level of GluR2 is low. Therefore, the PC12 cell line is a good model for our present study. As anticipated from our previous data in *Rsk2*-KO neurons [9], silencing of the *Rsk2* gene led to an up-regulation of ERK1/2 activity and to an increase of GluR2 expression, both being, however, stronger than those observed in *Rsk2*-KO neurons [8,9]. Increase of GluR2 expression was observed at both the protein and mRNA levels, indicating that it was predominantly the transcription of the *Gria2* gene that was affected by the absence of RSK2 activity. Since it was previously reported that the ERK pathway plays a role in the regulation of GluR2 expression [27], we hypothesized that the increased GluR2 expression level in the *Rsk2*-KO mice and in *Rsk2* knockdown PC12 cells might be related to the elevated ERK1/2 activity. As expected, inhibition of ERK1/2 activity in the *Rsk2* knockdown PC12 cells with the U0126 inhibitor reversed the effect of RSK2 depletion on GluR2 expression, clearly implicating ERK1/2 in the elevated GluR2 expression.

Previous investigations demonstrated a positive regulatory role for Sp1 in the transcriptional regulation of *Gria2* gene via cis-acting elements in its proximal promoter regions [11]. Multiple signaling pathways converge on Sp1, including ERK, Akt, and c-Jun *N*-terminal protein kinase [28]. ERK-regulated phosphorylation sites on Sp1 were identified at T453 and T739 in response to growth factor regulation of the vascular endothelial growth factor gene [29]. Phosphorylation of Sp1 on T453 and T739 increases Sp1 DNA binding activity [29]. Our data show that levels of phosphorylation at both Sp1 sites is strongly increased in *Rsk2* knockdown PC12 cells, whereas treatment with the MEK inhibitor U0126 inhibits phosphorylation at both of these sites, and also abolishes the increased level of GluR2 expression in RSK2 deficient cells. Thus, our results confirm the implication of ERK1/2 in Sp1 phosphorylation at both T453 and T739 Sp1 sites, and indicate that the increased levels of Sp1 phosphorylation and GluR2 expression in RSK2 deficient cells are related to the elevated ERK1/2 activity. Finally, the evidence that a significant reduction of Sp1 expression, with a Sp1-specific siRNA, prevents the increased level of GluR2 expression in RSK2 deficient cells further supports the critical role of Sp1 in the up regulation of GluR2 expression. How phosphorylated Sp1 activates the *Gria2* promoter remains now to be investigated. It is possible that Sp1 phosphorylation may change its interaction with other transcription factors. Sp1 phosphorylation induced by the ERK pathway was also shown to cause the release of a histone deacetylase co-repressor complex from a gene promoter [30].

Previously, it was shown that changes in the expression levels of RSK2 can modify both the expression levels and the subcellular distribution of GluR2 subunits [8]. Our present data, by showing that RSK2 can modulate indirectly GluR2 expression by increasing or decreasing ERK1/2 activity, provides a potential mechanism for these modifications. RSK2 was also shown previously to directly interact with GluR2 [31]. Together the data provide increasing evidence that RSK2 plays an important role in the regulation of the function of AMPA receptors.

*Gria1* (encoding GluR1) and *Gria2* promoters share common features, such as multiple transcription start sites, GC rich, lack of a TATA box, and presence of Sp1 binding site *in vitro* [11,32]. However, RSK2 deficiency causes only an enhancement of GluR2 but not GluR1 or GluR3 protein expression levels both in hippocampal neurons [8] and PC12 cells (present study), which further underscores the specific role of the GluR2 AMPA receptor subunit. In the present study, the expression of GluR1 is even slightly down regulated in RSK2 depleted PC12 cells. Our data provide evidence that the regulation of *Gria1* and *Gria3* genes expression might be under the control of distinct signals that remain to be identified.

The vast majority of male patients affected with Coffin-Lowry syndrome have severe-to-profound intellectual disability, and currently there is no cure for this disorder. Our finding of an abnormally increased level of GluR2 expression in RSK2 deficient neurons could lead to therapeutic strategies for CLS. Indeed, in the Fragile X syndrome (FXS), observations suggesting increased metabotropic glutamate receptor (mGluR5) expression led to preclinical studies showing that the inhibition of mGluR5 can ameliorate multiple mutant phenotypes in mouse and drosophila models of FXS, and clinical trials based on this therapeutic strategy are underway [33]. Modulation of GluR2 levels may be a useful pharmaceutical approach for improving cognitive function. However, it is first necessary to validate the mechanism described in the present report in *Rsk2*-KO mice, and the present results in PC12 cells will facilitate this validation. This will need the use of lentiviral mediated gene and ShRNA transfer, and preliminary experiments have been undertaken. It is also essential to further dissect the molecular mechanism of *Gria2* transcriptional control, including the identification by which Sp1 activates the *Gria2* promoter. In addition to Sp1, *Gria2* expression is also influenced at the transcriptional level by another positive (Nrf-1) and a negative (Re1/Nrse-like silencer) regulatory element in the 5′ proximal region of the promoter [11]. Myers *et al*. [32] showed that the *Gria2* Re1/Nrse-like silencer binds the RE1-silencing transcription factor (NRSF/REST), and that co-transfection of REST into neurons reduced *Gria2* promoter activity in a silencer-dependent manner. The nuclear respiratory factor 1 (NRF-1) is a transcription factor that was also shown to regulate *Gria2* gene transcription in neuroblastoma cells [34]. It is clearly necessary to understand the roles of the different regulatory elements and their interplay in neurons. In addition, although there is evidence that rapid (AMPAR) excitatory synaptic transmission is affected in *Rsk2* mutant mice [8], the extent of glutamate transmission alteration, and whether synaptic plasticity is modified, remain to be explored. These studies will provide more insights into the feasibility of targeting *Gria2* transcription in Coffin-Lowry syndrome treatment.

## 4. Materials and Methods

### 4.1. Cell Culture

PC12 cells were grown on Dulbecco’s Modified Eagle Medium (DMEM) medium (glucose 4.5 g/L) (Gibco by Invitrogen, Carlsbad, CA, USA) supplemented with 10% horse serum and 5% fetal bovine serum. To induce terminal differentiation, we added 50 ng/mL Nerve Growth Factor-β (NGF), (Chemicon Millipore, Temecula, CA, USA). NGF was supplied for a minimum of 4–5 days before transfection.

### 4.2. Transfection of PC12 Cells with shRNA Vectors

We first tested two mouse shRNAs (SureSilencing, Qiagen S.A. Courtaboeuf, France) cells. We then tested a shRNA directed against the human *Rsk2* gene (according to the nomenclature: *RPS6KA3* gene) (SureSilencing TM shRNA Plasmids for Human *RPS6KA3*, Clone ID7) and matching completely with the rat *Rsk2* gene, which decreased *Rsk2* expression dramatically. Thus, the shRNA clone ID7 was used in all subsequent studies (and named *Rsk2*-shRNA). Approximately 10^3^ to 10^4^ undifferentiated cells were plated in each of six well plates. After differentiation the cells were used for transfection. Briefly, 5 μg of *Rsk2*- or scrambled-shRNA, diluted in Opti-MEM^®^ I, were added to each well and transfection was performed with Lipofectamine 2000 according to the manufacturer’s instructions. Cells were incubated for 6 h at 37 °C in a CO_2_ incubator, then the medium was changed for fresh medium and the plates stored again in the incubator. After 24 h of transfection, G418 (0.6 mg/mL) (Gibco by Invitrogen) was added to the medium and the plates again stored in the incubator.

### 4.3. siRNA Transfection

Untransfected and shRNA transfected PC12 cells were used 24 h after transfection for a second transfection to silence the Sp1 gene expression. The complex of siRNA (Sp1-siRNA: sc-61895, Santa Cruz Biotechnology, Dallas, TX, USA) and Lipofectamine^®^ RNAiMAX in Opti-MEM^®^ I (both from Invitrogen, Carlsbad, CA, USA) was prepared according to the manufacturer’s instructions. After this second transfection cells were incubated for 48 h at 37 °C in a CO_2_ incubator, and then proteins were extracted using the RIPA buffer (Santa Cruz Biotechnology) (150–200 μL/well).

### 4.4. Cell Cultures Treatment with the MEK Inhibitor

U0126 (20 mM) (9903; Cell Signaling Technology, Beverly, MA, USA) was added to the culture medium of shRNA transfected PC12 cells four days after transfection and stored at 37 °C in the incubator. Twenty-four hours later proteins were extracted as above.

### 4.5. Western Blot Analysis

Western blot analyses were performed as previously described [35]. Thirty μg of protein extracts for each sample were loaded on the SDS-PAGE gel. After scanning of the autoradiography films, quantifications were carried out with the GeneTool software of the Chemigenius apparatus (Syngene, Cambridge, UK) and results were normalized to the level of the housekeeping protein GAPDH.

The following antibodies were used: anti-ERK (9102), anti-P-ERK (9106), anti-RSK1 (9333) (all three from Cell Signaling Technology, Beverly, MA, USA), anti-RSK2 (sc-1430) and anti-RSK3 (sc-1431) (both from Santa Cruz Biotechnology), GluR2 (AB 1768-25UG), Sp1 (07-645), p-Sp1 (T453, ab37707), p-Sp1(T739, BS4755) and glyceraldehyde-3-phosphate dehydrogenase (*Gapdh*, MAB374) (all five from Millipore Corporation, Bedford, MA, USA).

### 4.6. Real-Time QRT-PCR Analysis

Total RNA from PC12 cultures was extracted using Tri-Reagent (TR-118, Molecular Research Center Inc., Cincinnati, OH, USA) according to the manufacturer’s instructions. Reverse transcription (RT) was performed on 1 μg RNA using the Transcriptor kit (03 531 317 001; Roche Molecular Biochemicals, Indianapolis, IN, USA) to generate cDNA with oligo dT following the manufacturer’s protocol. Real time QRT-PCR was performed with LightCycler480 Sybr Green I Master (Roche SA, Boulogne-Billancourt, France) and achieved using a Light Cycler instrument (Roche) [35]. The sequence of PCR primers used for detection of *Rsk2* (Forward 5′-ACAAGGGGTGGTTCACAGAG-3′, Reverse 5′-GCATCATAACCTTGCCGTTT-3′), Gria2 (Forward 5′-TTTCCTTGGGTGCCTTTATG-3′, Reverse 5′-GACAGATCCTCAGCACTTTCG-3′) and Sp1 (Forward 5′-CAGACTCAGTATGTGGACCAA-3′, Reverse 5′-GTTGAATAGCTGTTGGCAT-3′). All results were normalized using quantification of the housekeeping gene *Gapdh* (Forward 5′-CCAAAAGGGTCATCATCTCC-3′, Reverse 5′-GAGGGGCCATCCACAGTCTT-3′).

### 4.7. Immunofluorescence

Untransfected and Transfected PC12 cells grown on slides were washed three times with 1X Phosphate Buffered Saline (PBS), fixed for 10min with 4% PFA and 2.5 M glycine and washed again 3 times (1X PBS). Slides were then immersed in PBS-Triton 0.1% for 15 min and rinsed again 3 times with PBS. The cells were then incubated with the primary monoclonal antibody (Goat-anti-RSK2, 1:500, Santa Cruz Biotechnology) overnight, followed by incubation with a fluorescence-conjugated secondary antibody for 1 h, and finally dehydrated and mounted with KAISER’s glycerol gelatin (Merck, Fontenay Sous Bois, France).

### 4.8. Statistical Analysis

All data are expressed as mean ± SEM and comparisons between groups were made by a Student’s *t*-test. Differences with *p* < 0.05 were considered significant.

## 5. Conclusions

Our findings suggest an indirect role of RSK2 in modulating the expression of AMPA receptor GluR2 subunit and, therefore, in regulating glutamatergic neurotransmission and probably synaptic plasticity. Future studies are warranted to elucidate the molecular mechanisms that underline the interplay between the *Rsk2* gene and the glutamatergic system.
